# Associations of fish oil with cardiovascular disease events: results from the Taiwan longitudinal study in aging

**DOI:** 10.1186/s12889-024-19512-8

**Published:** 2024-07-24

**Authors:** Hsiu-Chuan Chen, Chi-Jung Tai, Jing-Yang Huang, Tsu-Ann Kuo, Yuan-Der Huang, Chi-Hua Yen, Meng-Chih Lee

**Affiliations:** 1 Department of Family Medicine, Taichung Hospital, Ministry of Health and Welfare, Taichung, Taiwan; 2 Department of Family Medicine, Kaohsiung Medical University Hospital, Kaohsiung, Taiwan; 3 Department of Family Medicine, Kaohsiung Medical University Gangshan Hospital, Kaohsiung, Taiwan; 4 Department of Family Medicine, School of Medicine, Kaohsiung Medical University, Kaohsiung, Taiwan; 5 Institute of Medicine, Chung Shan Medical University, Taichung, Taiwan; 6 Department of Medical Research, Chung Shan Medical University Hospital, Taichung, Taiwan; 7 Department of Medical Sociology and Social Work, Chung Shan Medical University, Taichung, Taiwan; 8 Department of Obstetrics and Gynecology, Taichung Hospital, Ministry of Health and Welfare, Taichung, Taiwan; 9 Min-Hwei Junior College of Health Care Management, Tainan, Taiwan; 10 School of Medicine, Chung Shan Medical University, Taichung, Taiwan; 11 Department of Family and Community Medicine, Chung Shan Medical University Hospital, Taichung, Taiwan; 12 College of Management, Chaoyang University of Technology, Taichung, Taiwan; 13 Institute of Population Health Sciences, National Health Research Institutes, Miaoli, Taiwan

**Keywords:** Fish oil, Cardiovascular event, Stroke, Longitudinal study, Diabetes

## Abstract

**Background:**

The effectiveness of fish oil in preventing cardiovascular events is still debating. Some studies indicate a correlation between the use of fish oil supplements and reduced mortality or decreased incidence of stroke. However, other studies show no significant association between fish oil intake and stroke prevention, indicating an ongoing debate. This study aimed at exploring which subjects may benefit more from fish oil supplementation.

**Methods:**

This study utilized the data obtained through face-to-face interview from the Taiwan Longitudinal Study in Aging (TLSA). A total of 3,652 participants were included from the 2003 baseline data, after excluding patients with pre-existing ischemic heart disease or stroke. Participants were divided into two groups based on whether taking fish oil supplement or not. Participants were followed until 2015, estimating and comparing the all-cause mortality and cumulative incidence rate of stroke between both groups.

**Results:**

The results of the 12-year longitudinal study showed that the cumulative incidence rate of stroke in the fish oil supplementation group was 5.7%, compared to 7.7% in the non-supplemented group (*P* < 0.05). Additionally, the crude hazard ratio for stroke was significantly lower in the fish oil supplementation group (HR = 0.686;95% CI 0.476–0.987). However, after adjusting potential confounders, the adjusted risk of stroke was lower only for the diabetic patients supplemented with fish oil (aHR = 0.123; 95% CI 0.016–0.930) compared to non-diabetic patients (aHR = 0.917; 95% CI 0.616–1.364).

**Conclusion:**

This study suggests that there is an association between fish oil supplementation and a lower cumulative incidence rate of subsequent stroke among diabetic patients.

## Background

Cardiovascular disease (CVD) including ischemic heart disease and stroke, have been a leading cause of death and disability worldwide. In recent years, there has been increasing efforts on the prevention and treatment of CVD. Identifying relevant mechanisms and factors for disease prevention would be very helpful. Currently, it is known that factors such as gender, age, diet, and comorbidities can all have an impact on cardiovascular health [1]. However, there is still no consensus on whether nutritional supplementation, especially fish oil supplements, can provide protective effects.

In the 1970s, it was discovered that the incidence of CVD among the Inuit, whose diet was primarily meat-based, was significantly lower [2]. Subsequent research found this to be related to their main dietary intake of fatty fish. Fish oil is rich in long-chain omega-3 fatty acids, with the main components of the unsaturated fatty acids eicosapentaenoic acid (EPA) and docosahexaenoic acid (DHA) [3]. Several studies have suggested that supplementing with omega-3 fatty acids can help reduce the risk of CVD [4–8]. Guidelines from the American Heart Association also recommend using omega-3 fatty acids for secondary prevention of coronary heart disease [9], as well as consuming fish or using omega-3 supplements for primary prevention of CVD [6, 10]. As people become more aware of the health benefits of fish oil, it is becoming increasingly common, that nowadays many individuals regularly use fish oil as a dietary supplement [11–13].

However, some results from clinical trials have shown no significant correlation between supplementing omega-3 fatty acids and the prevention of CVD [14–16]. Additionally, studies on the association between supplementing omega-3 fatty acids and the risk of mortality have yielded inconsistent results. One trial study examining the impact of weekly fish consumption on heart disease risk found a significant reduction in overall mortality [17]. However, subsequent findings from long-term evaluation drew different conclusions, indicating an increased risk of coronary heart disease mortality in men with angina who consumed fish or fish oil supplements [18]. Several subsequent large trials studies have reported conflicting results between the omega-3 supplement and placebo groups in terms of cardiovascular events [19–23], rendering us unable to draw consistent conclusions and indicating the need for further research.

The current literature indeed shows inconsistent conclusions which is due to the different study design, which is why we aim to conduct our study using the Taiwan Longitudinal Study on Aging (TLSA) data. This longitudinal and national study should allow us to possibly draw more definitive conclusions. TLSA is a long-term and nationally representative database. In this study we aim to utilize data from the TLSA to explore the association between fish oil supplementation and the risk of cardiovascular events and related all-cause mortality.

## Methods

### Data source

The data for this study were obtained from the TLSA, a longitudinal study conducted by the Health Promotion Administration, Ministry of Health and Welfare, Taiwan in collaboration with the Population Studies Center and the Institute of Gerontology of the University of Michigan in the United States. The TLSA aims to observe the health and living conditions of middle-aged and elderly individuals in Taiwan over an extended period. The first wave of TLSA data collection began in 1989, followed by subsequent follow-ups every 3 to 4 years. All personal interviews were conducted by highly trained interviewers under careful supervision and following strict interview guidelines to ensure quality. Nine study waves have been initiated, including 1989, 1993, 1996, 1999, 2003, 2007, 2011, 2015 and 2019. The TLSA employs a three-stage stratified random sampling method. Initially, Taiwan’s 331 townships and urban areas are divided into 27 strata based on administrative region, education level, and total fertility rate, and 56 townships and districts are randomly chosen. In the second stage, neighborhoods are selected in proportion to the sample population within these townships and districts. Finally, in the third stage, two elderly individuals are randomly selected from each neighborhood as sample cases. All samples in this study consist of individuals aged 50 and above. This study used well-established 4 waves’ survey data: 2003, 2007, 2011 and 2015. [24, 25].

The study sample is consisted of 4,382 individuals aged 50 and above who participated in both the 2003 and 2007 waves of the TLSA. Starting from the 2003 wave, we specifically asked participants if they had a history of ischemic heart disease or stroke and followed up by asking if they had been diagnosed by a physician. This part was conducted through face-to-face interviews by well-trained interviewers who adhered to the questionnaire guidelines to make clear the questions and answers. We ensured that the respondents understood the definitions of the diseases, so we believe that the answers we obtained in the 2003 wave accurately reflected the presence of these conditions in the excluded participants. In the other word, all those free of CVD diseases or stroke at the starting point were included in this follow-up study to avoid the selection bias.

**Fig. 1:**
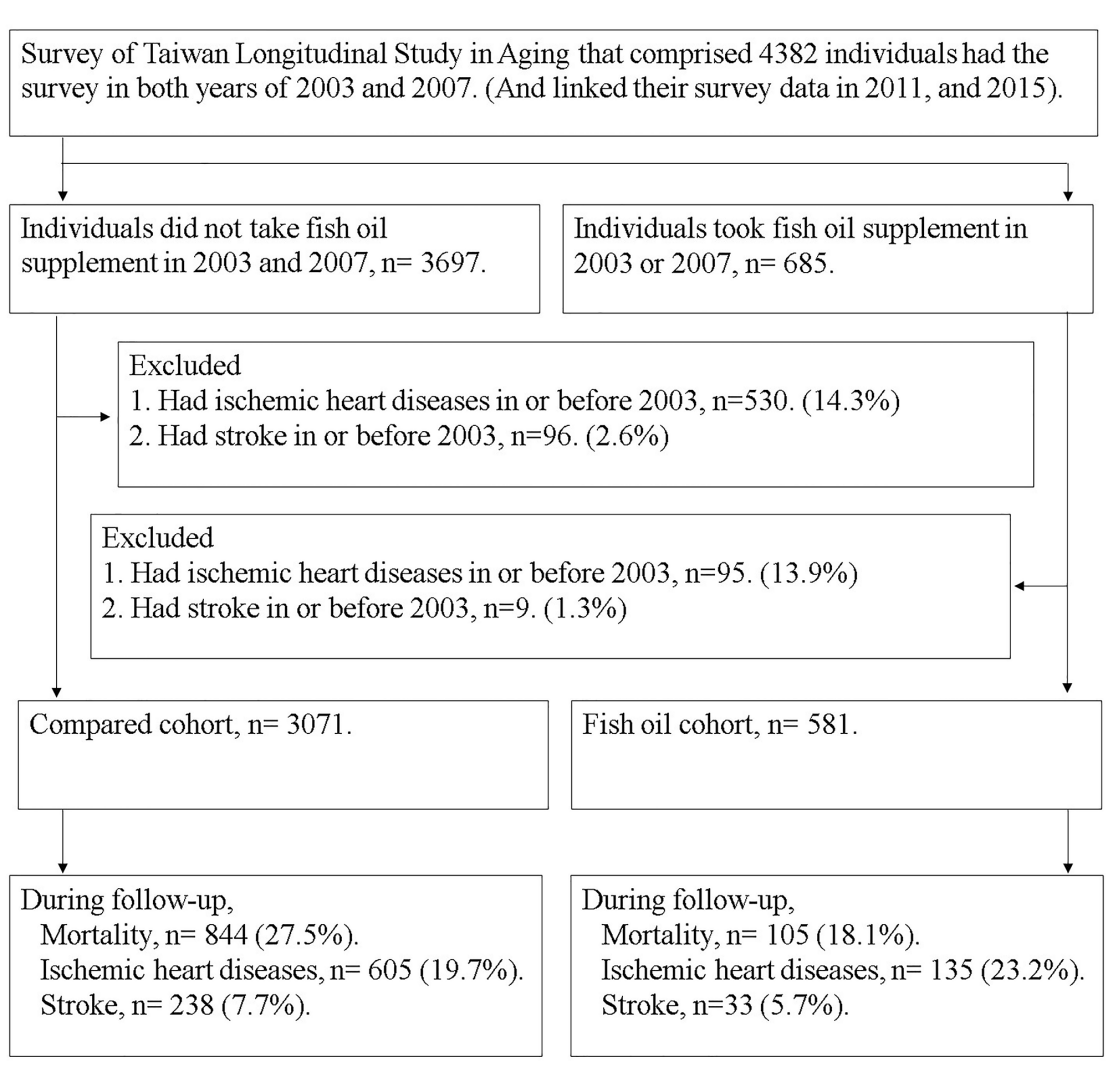
Study flowchart. All study individuals were follow-up since the year of 2003 until loss to follow-up, death or end in the year of 2015

After excluding individuals diagnosed with ischemic heart disease or stroke by physicians at baseline, 3,652 participants were included in the study. They were divided into two groups: those who were taking fish oil supplements (*N* = 581) and those who had never taken fish oil supplements (*N* = 3,071). Participants were followed up until 2015 to analyze the effect of fish oil supplement ischemic heart disease and stroke. Additionally, the mortality data from the Ministry of Health and Welfare for the year 2015 were linked to analyze the association between fish oil supplementation and mortality (Fig. 1).

### Independent variable

The use of fish oil was the independent variable. In the 2003 and 2007 questionnaires, participants were asked, “Do you usually use or take fish oil?” Those who answered “No” were categorized into the non-fish oil supplementation group, while those who answered “Yes” including continued or intermittent use were categorized into the fish oil supplementation group.

### Dependent variables

The dependent variables included information collected from the questionnaire on stroke and ischemic heart disease in 2015. Participants were asked, “Do you have ischemic heart disease or stroke?” If they answered “No” or “I don’t know,” they were classified as not having these conditions. If they answered “Yes,” they were further asked, “Has this disease been diagnosed by a doctor?” Participants who answered “Yes” were classified as having these conditions. Furthermore, data from the 2015 Taiwan National Death Registry (TNDR) were used to examine the mortality rate among the participants.

### Covariates

The literature reports that age [26, 27], gender [28], BMI [29], smoking [30–32], and alcohol consumption [33] are significantly associated with the risk of cardiovascular diseases. Additionally, level of education and income are associated with CVD outcomes, with higher education levels and higher income groups having lower cardiovascular disease risk [34, 35]. Furthermore, participants with better perceived health status may have healthier behaviors, including maintaining good lifestyle habits such as regular exercise and fewer unhealthy habits. Chronic diseases (DM, hypertension, cancer) are also associated with CVD outcomes, with diabetic patients having a significantly increased risk of cardiovascular diseases [36]. Based on the above, those covariates are included in this study.

Age was categorized into four groups: 50–59 years, 60–69 years, 70–79 years, and ≥ 80 years. Education level was classified into ≤ 6y (illiteracy and elementary school), 7-12y (junior high school to senior high education) and 13 + y (college degree or above). Perceived health status and economic status were evaluated for each individual and categorised into three groups: very good/good, fair, or poor/very poor. Additionally, participants’ reported exercise habits were classified into without exercise, 1–2 times, 3–5 times and ≥ 6 times per week.

### Statistical analyses

Descriptive statistics were used for demographic distribution, and the Chi-square test was applied for categorical variables. Kaplan-Meier survival analysis curves were used to estimate the all-cause mortality rate from 2003 to 2015 and the cumulative incidence rates of ischemic heart disease and stroke in both groups. The log-rank test was used to assess the differences in cumulative stroke incidence rates between the two groups. Cox regression analysis was conducted to estimate the crude hazard ratio and adjusted hazard ratio of fish oil supplementation on the stroke occurrence. Cox regression analysis was used to examine the odds ratio of developing stroke after taking fish oil supplementation for specific chronic diseases, controlling for age, gender, BMI, levels of education, perceived health status, economic status, exercise habits, smoking, alcohol consumption, and chronic disease status (hypertension, diabetes mellitus, and cancer). The impact of fish oil supplementation on stroke was further explored.

The significance level for statistical analysis was set at 0.05. SAS Software 9.4 was used for all the statistical analysis and data processing.

## Results

A total of 3,652 participants met the criteria for this study, with 3,071 in the fish oil supplementation group and 581 in the non-supplementation group. The follow-up period had been lasted for 12 years, with a minimum of 4 years and a maximum of 12 years. Chi-square tests were used to analyze the characteristics of the two groups. The results indicated that participants in the fish oil supplementation group, selected at the starting point in 2003, were younger, had higher levels of education, perceived better health status, better economic conditions, engaged in more weekly exercise session, and less likely to smoke. However, there were no significant differences in gender, BMI, alcohol consumption, and comorbidities between the two groups (Table 1).

**Table 1:** The baseline characteristic among the individuals used fish oil supplement (fish oil cohort) and the compared cohort

	Compared cohort	Fish oil cohort	*p* value
n	3071	581	
**Baseline characteristic**			
Age			0.0006
50–59	1309 (42.6%)	274 (47.2%)	
60–69	762 (24.8%)	166 (28.6%)	
70–79	725 (23.6%)	110 (18.9%)	
80+	275 (9.0%)	31 (5.3%)	
Sex			0.0840
Male	1600 (52.1%)	280 (48.2%)	
Female	1471 (47.9%)	301 (51.8%)	
BMI Missing	112 (3.6%)	7 (1.2%)	
BMI (Mean$\:\pm\:$SD)	24.08 ± 3.47	24.30 ± 3.63	0.1604
Education			< 0.0001
≤ 6 y	2132 (69.4%)	288 (49.6%)	
7–12 y	687 (22.4%)	185 (31.8%)	
13 + y	252 (8.2%)	108 (18.6%)	
Perceived health status			0.0074
Good/Very good	1464 (47.7%)	289 (49.7%)	
Fair	949 (30.9%)	200 (34.4%)	
Poor/Very poor	658 (21.4%)	92 (15.8%)	
Economic status			< 0.0001
Good/Very good	1135 (37.0%)	265 (45.6%)	
Fair	996 (32.4%)	199 (34.3%)	
Poor/Very poor	839 (27.3%)	108 (18.6%)	
Missing or unknown	101 (3.3%)	9 (1.6%)	
Exercise			< 0.0001
Without exercise	1136 (37.0%)	146 (25.1%)	
1–2 times per week	269 (8.8%)	65 (11.2%)	
3–5 times per week	341 (11.1%)	83 (14.3%)	
≥ 6 times per week	1325 (43.2%)	287 (49.4%)	
Smoking	731 (23.8%)	100 (17.2%)	0.0005
Alcohol	1002 (32.6%)	206 (35.5%)	0.1839
Co-morbidity			
Hypertension	719 (23.4%)	152 (26.2%)	0.1539
Diabetes mellitus	310 (10.1%)	60 (10.3%)	0.8647
Cancer	44 (1.4%)	13 (2.2%)	0.1513

**Fig. 2:**
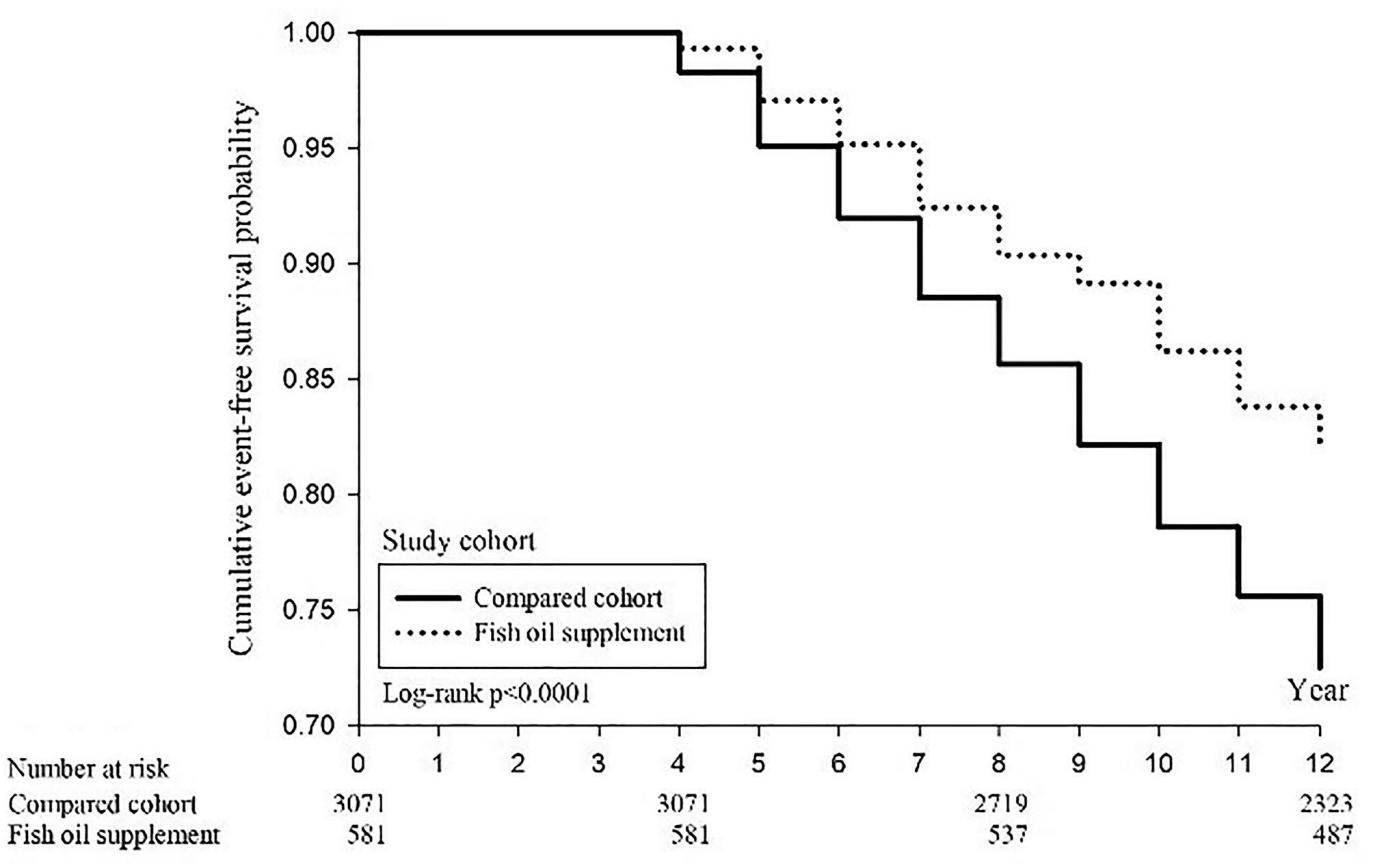
Kaplan–Meier curves of cumulative survival curve between fish oil supplement and compared group

**Fig. 3:**
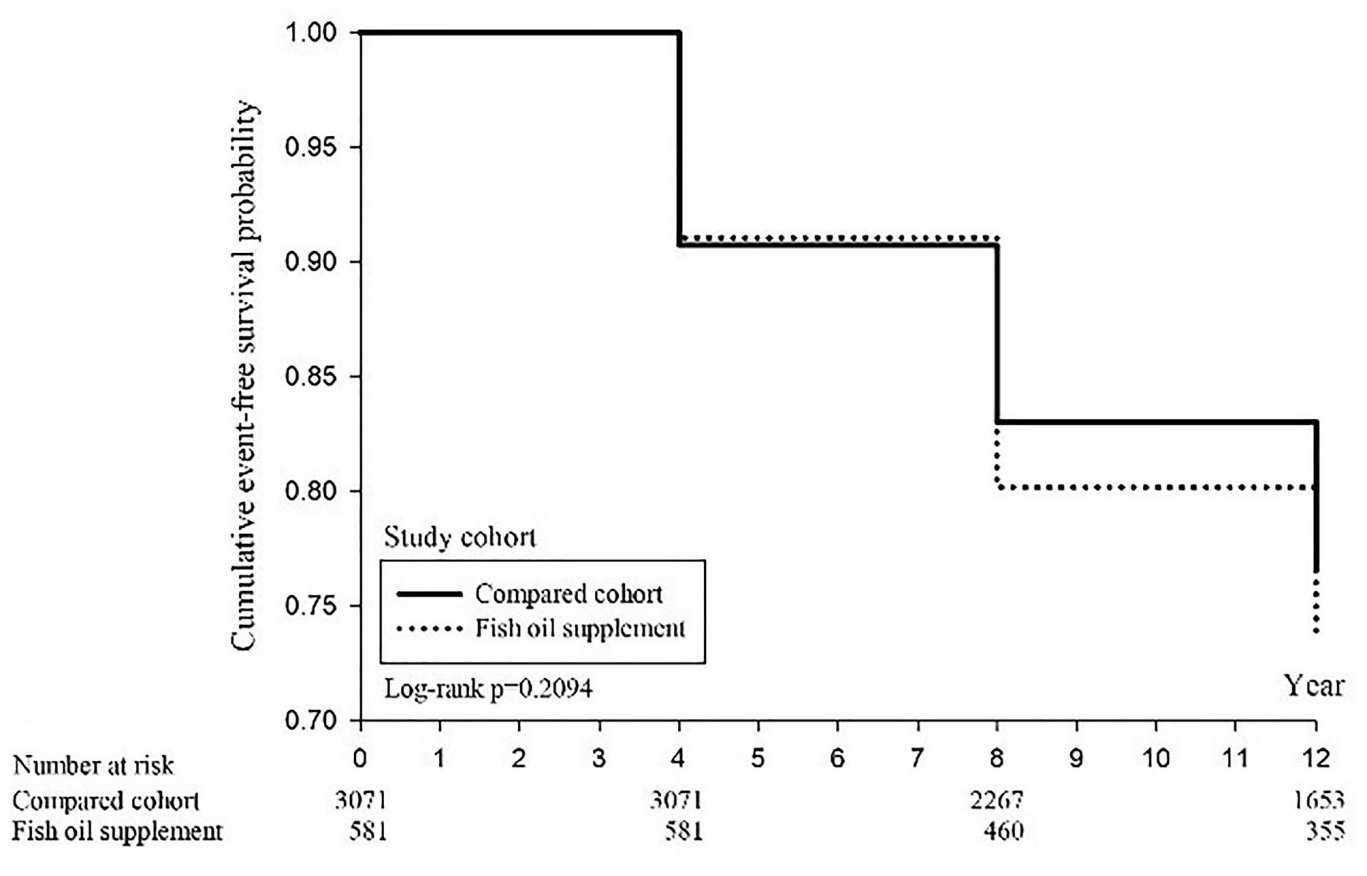
Kaplan–Meier curves of cumulative ischemic heart disease-free rate between fish oil supplement and compared group

**Fig. 4:**
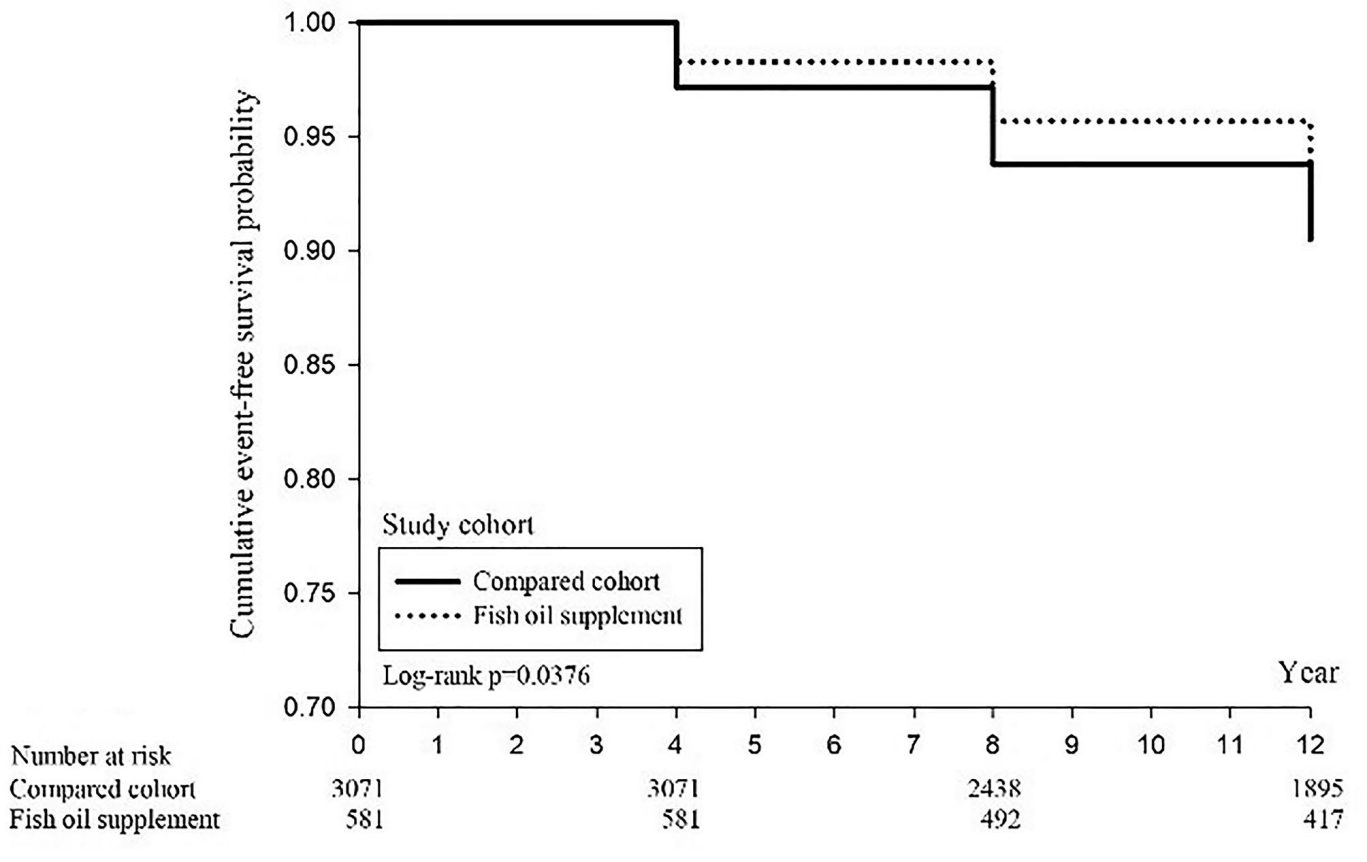
Kaplan–Meier curves of cumulative stroke-free rate between fish oil supplement and compared group

The Kaplan-Meier (KM) survival curves for the 12-year follow-up of the fish oil supplementation and non-lementation groups show a higher cumulative survival rate in the fish oil supplementation group (81.9%) compared to the non-supplementation group (72.5%) (Fig. 2). The log-rank test indicates a significant difference in the trend of mortality between the two groups (*P* < 0.0001). Additionally, there was no significant difference in the cumulative incidence rate of ischemic heart disease between the two groups (Fig. 3). The cumulative incidence rate of stroke in the fish oil supplementation group was 5.7%, compared to 7.7% in the non-supplementation group, demonstrating a significantly lower rate of stroke in the fish oil supplementation group (*P* = 0.0376) (Fig. 4).

Table 2 presents the risk associated with fish oil supplementation for specific outcomes. Firstly, Moreover, fish oil supplementation was associated with a reduction in mortality risk of approximately 16.5%. However, the adjusted hazard ratio (aHR) showed no significant difference (aHR = 0.835; 95% CI 0.676–1.032). Secondly, there was no significant difference in the risk of ischemic heart disease between the fish oil supplementation group and the non-supplementation group, whether in crude hazard ratio (HR = 1.121; 95% CI 0.930–1.351) or adjusted hazard ratio (aHR = 1.066; 95% CI 0.877–1.296). Third, the crude hazard ratio for stroke showed a significant difference between the groups (HR = 0.686; 95% CI 0.476–0.987), the adjusted hazard ratio for stroke indicated no significant difference (aHR = 0.786; 95% CI 0.536–1.152) (Table 2).

**Table 2:** The risk of all-cause mortality, ischemic heart disease and stroke in study cohorts

	Compared cohort	Fish oil cohort
N	3071	581
Risk of all-cause mortality		
Followed person-years	21,374	4262
Mortality event	844	105
Incidence density* (95% CI)	3.95 (3.69–4.22)	2.46 (2.02–2.98)
Crude hazard ratio (95% CI)	Reference	0.624 (0.510–0.765)
Adjusted hazard ratio (95% CI)	Reference	0.835 (0.676–1.032)
Risk of ischemic heart disease		
Followed person-years	27,964	5584
Ischemic heart disease event	605	135
Incidence density (95% CI)	2.16 (2.00-2.34)	2.42 (2.03–2.86)
Crude hazard ratio (95% CI)	Reference	1.121 (0.930–1.351)
Adjusted hazard ratio (95% CI)	Reference	1.066 (0.877–1.296)
Risk of stroke		
Followed person-years	29,616	5960
Stroke event	238	33
Incidence density (95% CI)	0.80 (0.70–0.91)	0.55 (0.38–0.78)
Crude hazard ratio (95% CI)	Reference	0.686 (0.476–0.987)
Adjusted hazard ratio (95% CI)	Reference	0.786 (0.536–1.152)

*Incidence density, per 100 person-years

**The adjusted hazard ratios were estimated by the multiple Cox regression including the baseline covariates of fish oil supplement, age, sex, BMI, education, perceived health status, economic status, exercise, smoking, alcohol, hypertension, diabetes mellitus, and cancer

**Fig. 5:**
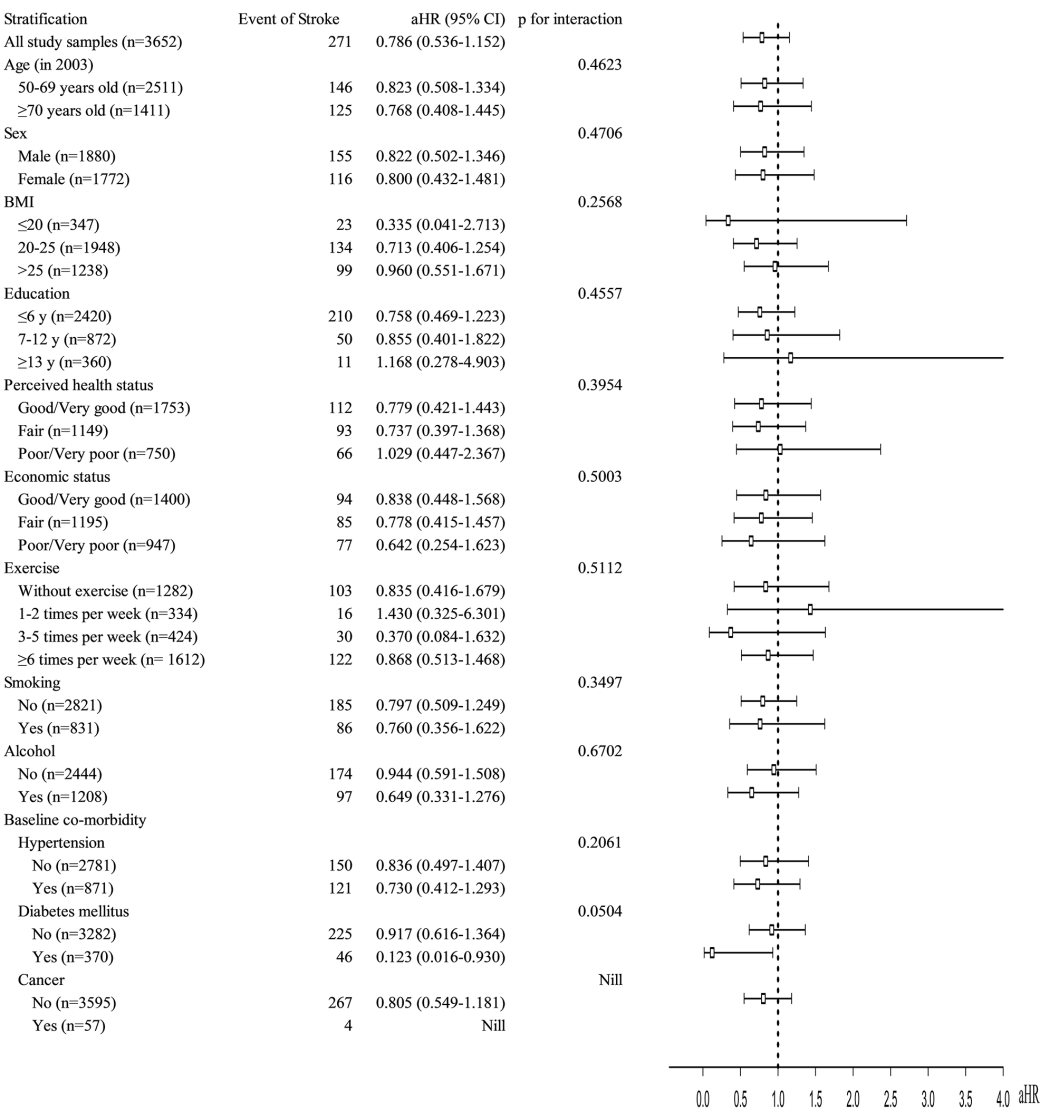
The baseline co-variate stratified analysis for the adjusted hazard ratio of stroke in fish oil supplement cohort compared with individuals without fish oil supplement. The adjusted hazard ratios were estimated by the multiple Cox regression including the covariates of baseline (year = 2003) age, sex, BMI, education, perceived health status, economic status, exercise, smoking, alcohol, hypertension, diabetes mellitus, and cancer

Furthermore, stratified analysis was conducted to explore which subgroups with chronic diseases may benefit more from fish oil supplementation (Fig. 5). The results suggest that there is a significant association between fish oil supplementation and a protective effect against stroke occurrence among diabetic patients. Specifically, the fish oil supplementation group among diabetic patients had a lower risk of stroke compared to the non-supplementation group (aHR = 0.123; 95% CI 0.016–0.930), with this adjusted hazard ratio being lower than that of non-diabetic patients (aHR = 0.917; 95% CI 0.616–1.364).

## Discussion

In Taiwan, cardiovascular-related diseases account for three of the top ten causes of death. In order to reduce the risk of CVD and stroke, dietary considerations should focus on high fiber, low sodium, low saturated fat, and low cholesterol intake, in addition to lifestyle modifications such as regular exercise and weight control. In recent years, there has been considerable attention on the role of omega-3 fatty acids in the prevention and improvement of ischemic heart disease, with dietary recommendations in many countries emphasizing the intake of omega-3 fatty acids [37].

Initially, we conducted the Kaplan-Meier (KM) analysis (Fig. 2) and found significant differences between the fish oil supplementation and non-supplementation groups, with the log-rank test also indicates significant differences. However, to be cautious, we used the multiple Cox regression to adjust for covariates to avoid their impact on survival rates. After controlling for those covariates, the significant difference disappeared. Due to the differences in baseline characteristics between two groups, the fish oil supplementation group initially exhibited a lower risk of all-cause mortality before adjustment. Considering the influence of potential confounding factors, our analysis using multiple Cox regression, which included key covariates in the analysis, indicated that fish oil supplementation was not associated with all-cause mortality. Thus, the identified covariates indeed had a confounding effect on the observed outcomes.

Evidently, there are confounding factors, including age, education level, perceived health status, economic status, smoking, and exercise. Younger participants may have better overall health and a lower baseline risk of cardiovascular events, which could partially explain the observed protective effect in the fish oil supplementation group. Higher levels of education may correlate with better health awareness and healthier lifestyle choices, potentially influencing the study outcomes. Additionally, participants with better perceived health status may be more proactive in adopting health-promoting behaviors, including fish oil supplementation. Better economic conditions may afford participants improved nutrition and healthcare. The fish oil supplementation group engaged in more weekly exercise sessions, contributing to better cardiovascular health. Finally, past literature has reported that smoking is a leading cause of cardiovascular morbidity and mortality [30–32], and our study results show lower rates of smoking in the fish oil supplementation group. Considering the baseline differences between the fish oil supplementation and non-supplementation groups, there could be some confounders affecting the outcomes. Therefore, we used multivariate regression to adjust for these potential confounders to mitigate their impact on the outcomes.

To reduce the risk of ischemic heart disease and stroke, for prevention it is not enough to rely solely on fish oil supplementation. Covariates also play a role, and it is necessary to to be considered. Nevertheless, we wanted to further investigate which group of subjects might be beneficial from fish oil supplementation after controlling for those potential confounding factors. Therefore, we conducted a stratified analysis (Fig. 5) and found that fish oil supplementation was associated with a lower hazard ratio of stroke in diabetic patients.

The results of our study suggest that there is an association between fish oil supplementation and reduced crude mortality rates, but there was no difference after adjustment, which differs from the literature [38]. This may be related to the frequency of fish oil supplementation among the study sample. Since we only grouped participants into those who did and did not use fish oil, the group using fish oil included individuals with lower supplementation frequencies. Past literature has often reported associations between habitual fish oil supplementation and reduced all-cause mortality (aHR = 0.87; 95% CI 0.83–0.90) and decreased risk of CVD mortality (aHR = 0.84; 95% CI 0.78–0.91), as well as lower rates of cardiovascular events and stroke. Therefore, future research directions may further explore the differences in the frequency of fish oil supplementation and the risk of mortality and cardiovascular events.

An article published in NEJM mentioned that supplementing with fish oil cannot reduce the incidence of CVD and cancer [14]. The primary reason is that CVD and cancer have numerous risk factors, and it is insufficient to prevent these conditions solely by supplementing with omega-3 fatty acids; other related confounding factors must also be controlled. We have discussed this aspect in our discussion section as well. However, this does not deter us from understanding the impact of fish oil on subgroups.

Additionally, previous study [38] has shown that compared to the incidence of CVD, supplementation with fish oil is more strongly associated with ischemic heart disease mortality. This suggests that fish oil supplementation may have a more pronounced effect on individuals who have already experienced cardiovascular events. However, this study could not confirm the causes of death among participants. Analyzing causes of death could be considered as a future research direction.

The literature indicates a protective effect of omega-3 fatty acids against cardiovascular events. A meta-analysis involving 402,127 participants revealed that an increased frequency of fish consumption was associated with a reduced risk of stroke, particularly for ischemic stroke [39]. Unfortunately, the TLSA questionnaire does not specifically inquire about dietary intake, so the TLSA cannot provide variables in this regard. However, it is true that fish consumption is related to omega-3 fatty acid intake. Future studies should indeed consider this aspect. Another longitudinal study analyzing 183,291 participants found that supplementation with high-dose omega-3 fatty acids was associated with a decreased risk of stroke, particularly for ischemic stroke [40]. While this study did not explicitly distinguish between hemorrhagic and ischemic strokes, future research could explore this direction.

The results of this study suggest that there is an association between fish oil supplementation rich in omega-3 fatty acids and a lower cumulative incidence rate of stroke, with a significant protective effect against stroke observed in the diabetic population (aHR = 0.123; 95% CI 0.016–0.930). These associations were independent of risk factors, including age, gender, BMI, education level, perceived health, economic status, exercise habits, smoking and alcohol habits, and comorbidities. In subgroups analysis, there were participants 3,282 without DM, and 370 with DM. Due to the wide confidence interval though is significant in hazard ratio indicates prediction instability. This indeed requires future studies through increasing the sample size or extend the follow-up period for further validation.

Consistent with our findings, the American Diabetes Association (ADA) suggests that individuals with diabetes consume omega-3 fatty acid-rich foods to prevent CVD [41]. Regarding the potential benefits of fish oil supplementation in diabetic patients, literature suggests that omega-3 fatty acids in fish oil can regulate lipid metabolism in diabetic patients, thereby preventing cardiovascular events [42]. The hypothesis that omega-3 fatty acid supplements confer cardiovascular protection is supported by biological plausibility. Evidence from laboratory studies, animal research, and small human trials investigating intermediate cardiovascular endpoints supports various mechanisms. These mechanisms include antithrombotic effects, reduction in triglyceride levels, lowering of blood pressure, anti-inflammatory effects, inhibition of atherosclerotic plaque growth, and decreased susceptibility to cardiac arrhythmias. Collectively, these effects potentially reduce cardiovascular risk in diabetic patients [43, 44]. A meta-analysis focusing on type 2 diabetes found that omega-3 fatty acid supplementation significantly reduces diastolic blood pressure [45]. Another clinical trial involving women with type 2 diabetes showed a significant decrease in blood pressure with omega-3 fatty acid supplementation. Furthermore, a long-term follow-up study analyzing data from the National Health and Nutrition Examination Survey (NHANES) revealed a negative correlation between sufficient omega-3 fatty acid supplementation and all-cause mortality in diabetic patients, suggesting that adequate omega-3 fatty acid supplementation helps prevent premature death in diabetic patients [46]. However, it was not clear whether fish oil intake could significantly reduces adjusted all-cause mortality risk or decreases mortality risk in diabetic patients, warranting further investigation. Nevertheless, in terms of other cardiovascular benefits, this study did find a significant protective effect of fish oil supplementation among diabetic patients against stroke events. This aspect could be further explored through in-depth research into the underlying mechanisms.

This study has several strengths. Firstly, the TLSA database is a population-based cohort study, providing a representative sample of the health and lifestyle of middle-aged and elderly individuals in Taiwan. Secondly, the study conducted a 12-year follow-up survey of participants and analyzed the results after adjusting for many important risk factors. Thirdly, this is the first study to use the TLSA database to investigate the relationship between fish oil use and mortality as well as cardiovascular events.

However, the study also has certain limitations. Firstly, the TLSA questionnaire did not address the types and exact amount of fish oil supplements, so it is not possible to know the dose and formulation of fish oil supplements taken by the participants. Secondly, the use of self-report questionnaires might have recall bias. To minimize the impact of recall bias in our study, we used well-trained interviewers following standardized guidelines during face-to-face interviews. These measures could provide accurate information about their response. Additionally, due to the inability to confirm the causes of death among participants, further analysis on the causes of death should be taken into consideration. Finally, subgroups analysis had a wider 95% confidence interval indicating a prediction instability. This indeed requires future studies through increasing the sample size or extend the follow-up period for further validation.

Overall, the protective effect of omega-3 fatty acids on CVD has been widely recognized in other ethnic populations, and it appears to have similar benefits in an Asian sample in Taiwan. Particularly, there is an association suggesting a reduction in the incidence of stroke in patients with diabetes. We recommend encouraging diabetic patients to consider omega-3 fatty acid supplementation as part of primary prevention for stroke.

## Conclusion

The study results suggest a significant association between fish oil supplementation and a reduction in the risk of stroke among diabetic patients. Our findings suggest that there is an association between fish oil supplementation and a lower incidence of stroke among diabetic patients. This association indicates a potential future preventive healthcare or clinical strategy. However, further research is needed to explore the mechanisms underlying this benefit, as well as to determine the optimal dosage and duration of fish oil supplementation required to achieve it.

## Data Availability

The datasets used and analyzed during the current study are not publicly available, but are available from the corresponding author on reasonable request with the permission of the Ministry of Health and Welfare, Taiwan.

## Abbreviations

TLSA: Taiwan Longitudinal Study on Aging
CVD: Cardiovascular disease
BMI: Body Mass Index
EPA: Eicosapentaenoic Acid
DHA: Docosahexaenoic Acid
